# Neutrophil phenotyping reflects injury severity when base excess and lactate remain normal

**DOI:** 10.1007/s00068-026-03285-3

**Published:** 2026-08-12

**Authors:** F. J. C. van Eerten, L. V. Duebel, E. J. de Fraiture, S. R. Brands, K. J. P. van Wessem, N. Vrisekoop, J. H. Venhuizen, M. C. H. de Groot, S. Haitjema, L. Koenderman, F. Hietbrink

**Affiliations:** 1 Department of Trauma Surgery, University Medical Center Utrecht, Utrecht, GA 3508 Netherlands; 2 Department of Respiratory Medicine and Center for Translational Immunology (CTI), University Medical Center Utrecht, Utrecht, The Netherlands; 3 Central Diagnostic Laboratory, University Medical Center Utrecht, Utrecht University, Utrecht, The Netherlands

**Keywords:** Inflammation, Injury severity, Neutrophil, Physiology, Trauma

## Abstract

**Introduction:**

Accurate assessment of injury severity is essential for optimal trauma care, as it dictates timing and intensity of interventional strategy. Conventional markers, such as base excess and lactate levels are usually borderline deranged in patients with midrange injury severity. This study explored whether determining circulating neutrophil phenotypes in these patients (ISS 9–35) might aid in the assessment of injury severity.

**Methods:**

A descriptive single-center cohort study of trauma patients *≥* 16 years between 2013 and 2023 was conducted. Data was collected from the Dutch National Trauma Registry and the Utrecht Patient-Oriented Database (< 10% missing values). Base excess, lactate levels and neutrophil phenotypes were routinely measured in emergency department blood samples. The dynamics of these parameters was analyzed in the context of increasing Injury Severity Scores (ISS).

**Results:**

807 patients were included, of whom 591 were classified as midrange severely injured. In these patients, banded neutrophils showed a greater fold increase between ISS 9 and 35 compared with base excess and lactate (6.7, 2.7, and 1.5, respectively). Furthermore, the number of banded neutrophils exceeded the upper interquartile range of controls at lower trauma severities compared to base excess or lactate (ISS 17 vs. 30 and 39, respectively).

**Discussion:**

Although base excess and lactate perform well as indicators of physiological derangement in severe trauma (ISS > 35), increased numbers of banded neutrophils are detectable at lower ISS levels and rise more rapidly than conventional chemistry markers in midrange severely injured patients. As such, neutrophil phenotyping can complement traditional physiological markers in injury burden assessment in midrange severely injured patients.

**Supplementary Information:**

The online version contains supplementary material available at 10.1007/s00068-026-03285-3.

## Introduction

Traumatic injuries account for a substantial part of world-wide mortality and morbidity [1]. Appropriate timing and extent of surgical interventions is essential to reduce mortality and complications. Return to physiology restoration is frequently prioritized over definitive anatomical repair in severely injured patients [2, 3]. In these compromised patients, a Damage Control Surgery (DCS) is the approach of choice over Early Total Care (ETC), resulting preferably in Early Appropriate Care [2, 3].

The distinction between DCS and ETC primarily concerns the timing and extent of early surgical intervention [4]. The DCS approach involves abbreviated procedures aimed at rapid hemorrhage control and initial temporary biomechanical stabilization, while ETC entails early definitive fixation with often more extensive surgical procedures [4, 5]. Minimizing the surgical burden in appropriately selected patients may reduce the risk of organ failure and inflammatory complications at a later stage, by preventing further exacerbation of their already deranged physiology [6, 7]. While in contrast early definitive fixation might favor reduced risk of local complications and promote early functional recovery.

Base excess and lactate levels are commonly used to quantify the burden of injury and differentiate patients who may benefit from DCS [8, 9]. A base excess of *≤* -4 mmol/L has been identified as independent predictor for mortality in trauma patients in numerous studies [10–13]. Previous studies found that lactate levels *≥* 2.5 mmol/L are also predictive of mortality, both in critically ill and trauma patients [10, 12, 14, 15]. Base excess *≤* -4 mmol/L and lactate levels *≥* 2.5 mmol/L generally reflect a high injury burden with physiological derangement, making the indication for DCS more straightforward [10–13]. In contrast, when these parameters show borderline aberrant values, the choice between DCS and ETC might be more complicated and additional information is warranted [6, 7].

Notably, the extent of injury burden is also reflected in the post-traumatic inflammatory cellular response [16, 17]. Tissue injury leads to the release of damage-associated molecular patterns (DAMPs), which trigger the innate immune system [16–19]. The extent of this activation is reflected in the release of specific neutrophil subsets: CD16^high^CD62L^high^, CD16^low^CD62L^high^, CD16^high^CD62L^low^, which can be measured by determination of the neutrophil phenotypic categories [19–21]. There is increasing recognition that the assessment of neutrophil phenotypes, especially the occurrence of banded neutrophils, may complement traditional markers in surgical decision-making [16–19, 22–24].

The addition of banded neutrophils may be particularly valuable in supporting surgical decision making in patients without clear derangement in base excess and lactate levels, such as in the midrange severely injured patients (ISS 9–35). Rather than predicting injury severity, mortality or complications, this study focused on the dynamics of base excess, lactate, and neutrophil phenotypes within a midrange severely injured trauma population [7].

## Methods

### Study design

This is a descriptive single-center cohort study, conducted between 2020 and 2022 in the University Medical Centre Utrecht, the Netherlands. Patients were retrospectively identified using the institutional data of the Dutch National Trauma Registry (DNTR) [25]. The study was conducted in accordance with applicable ethics and privacy guidelines and was reviewed by the Ethics Review Committee (reference: 24U-0097_QoTc and 21-1016_AQUIretro2).

### Patient selection

All patients *≥* 16 years old, who were admitted between 2020 and 2022 to the hospital after sustaining a traumatic injury, with arterial blood gas analyses data available on initial base excess, lactate levels and neutrophil phenotyping at the Emergency Department (ED) were included. Data on base excess and lactate levels were extracted from the Utrecht Patient Oriented Database (UPOD) [26]. Experienced data managers followed an ISO-9001 certified protocol to obtain the data. Neutrophil phenotyping was performed as elaborated on below.

### Neutrophil phenotype assessment

Blood samples were obtained from patients in the trauma bay using 4 mL VACUETTE^®^ sodium heparin tubes (Greiner Bio-One, Austria). Sample processing followed the protocol outlined by Spijkerman et al. [27, 28] Tubes were loaded within 30 min into cassettes and inserted into the AQUIOS CL “Load & Go” flow cytometer (Beckman Coulter, CA, USA) for fully automated analysis. Before sample aspiration into a 96-well plate, gentle inversion of the tubes was performed by the device. A five-color antibody panel, including CD16-FITC (clone 3G8), CD11b (clone Bear1), CD62L (clone DREG-56), CD10 (clone ALB1), and CD64 (clone 22), all obtained from Beckman Coulter (CA, USA), was added and incubated for 15 min. Red blood cells were lysed using AQUIOS Lysing Reagent A (Beckman Coulter, CA, USA), with the reaction terminated after 30 s by AQUIOS Lysing Reagent B. Prepared samples were then aspirated into the flow cell for cytometric analysis, and output results were saved as raw data, which is loaded and analyzed in FlowSOM [29].

Moreover, the number of banded neutrophils was quantified using FlowSOM [29], a validated clustering algorithm for high-dimensional flow cytometry data. FlowSOM creates self-organizing maps to classify single-cell data and consistently identified three neutrophil metaclusters in LPS-stimulated control cells and trauma samples: CD16^brigh^t/CD62L^bright^ mature (MC1), CD16^dim^/CD62L^bright^ banded (MC2), and CD16^bright^/CD62L^dim^ hypersegmented (MC3) neutrophils. This approach was used because routine hematology analyzers do not reliably distinguish or recognize banded neutrophils.

### Data collection

Data was collected using the DNTR and UPOD [25, 26], by experienced data managers following an ISO-9001 certified protocol. Data collected included: age, sex, Glasgow Coma Scale (GCS), Abbreviated Injury Scale (AIS), systolic blood pressure (first measurement at ED), administered blood products in the first 24 h after admission (yes/no), lactate levels, base excess, and pH (first measurement at ED), number banded neutrophils (MC2), Injury Severity Score (ISS), length of hospital stay, length of ICU stay, in-hospital infectious complications and mortality. The ISS is retrospectively calculated as the sum of the squares of the AIS scores of the three most severely injured body regions [30, 31]. The AIS grades the severity of injuries within each body region [31]. Midrange injury severity was defined as ISS 9–35. Hemodynamic instability was defined as a systolic blood pressure below 90 mmHg [15]. Infectious complications predominantly comprised respiratory tract infections, urinary tract infections, and bloodstream infections, with smaller numbers of secondary meningitis, intra-abdominal infections, and osteosynthesis material infections. Wound and surgical site infections were not included. Infectious complications were included only if they developed following hospital admission, were diagnosed by a clinician, and prompted initiation of targeted treatment.

### Statistical analysis

The statistical analyses were performed using R for statistical computing (version 2024.12.0.46) [32]. Continuous variables are presented as medians with interquartile ranges (IQR). Categorical numbers were summarized as counts and percentages.

To evaluate the dynamics of base excess, lactate levels and banded neutrophils with increasing ISS, these parameters were plotted against the ISS. This approach allowed visualization of their respective trends across increasing injury burden, with specific attention to the midrange severity group (ISS 9–35). As this study focused on midrange severely injured patients, the patients with an ISS > 50 (7 patients) were excluded from the illustration. Visualization was performed using a Locally Estimated Scatterplot Smoothing (LOESS) line [33].

To illustrate when parameter levels diverged from control values, the median and IQR of control patients with minimal injury severity (ISS *≤* 3) were used as reference. This control cohort was selected to represent the baseline ED population without substantial traumatic injuries. To prevent confounding of physiological parameters in this control group, patients with injuries secondary to non-traumatic events (e.g., falls following seizures or cardiovascular incidents) were excluded from the control group.

Banded neutrophils, base excess and lactate levels were visualized across increasing ISS to show at which injury severity they exceeded the upper range of control values.

The associations between ISS and banded neutrophils, base excess and lactate levels were further assessed using Spearman’s rank correlation coefficient (ρ), due to potential non-linear relationships and non-normally distributed variables.

Subsequently, the total fold increase in case of banded neutrophils and lactate, and decrease in case of base excess, was calculated in the patients with an ISS between 9 and 35. Firstly, the median values of banded neutrophils, base excess and lactate per ISS scores were calculated for ISS 9 and ISS 35. Subsequently, fold increases or decrease in parameters were calculated by dividing the level at ISS 35 by the level at ISS 9 (Y_35_/Y_9_). Because base excess values surpass the zero-line, an offset of 2 was added to both Y_35_ and Y_9_ for this parameter to ensure the fold decrease could be accurately calculated.

Lastly, multivariable linear regression models were constructed to assess the independent association between banded neutrophils and hospital length of stay and ICU length of stay. A logistic regression was performed to investigate the association between banded neutrophils and infectious complications and 30-days mortality. All analyses were adjusted for age, sex and ISS. Neutrophil counts were log-transformed prior to analysis due to a highly skewed distribution. Generalized additive models (GAMs) were used in the linear regression models. Results were presented as β-coefficients in the linear regressions, or as odds ratios in the logistic regression, together with corresponding standard error or 95% confidence intervals and p-values.

## Results

### Patient selection

**Fig. 1:**
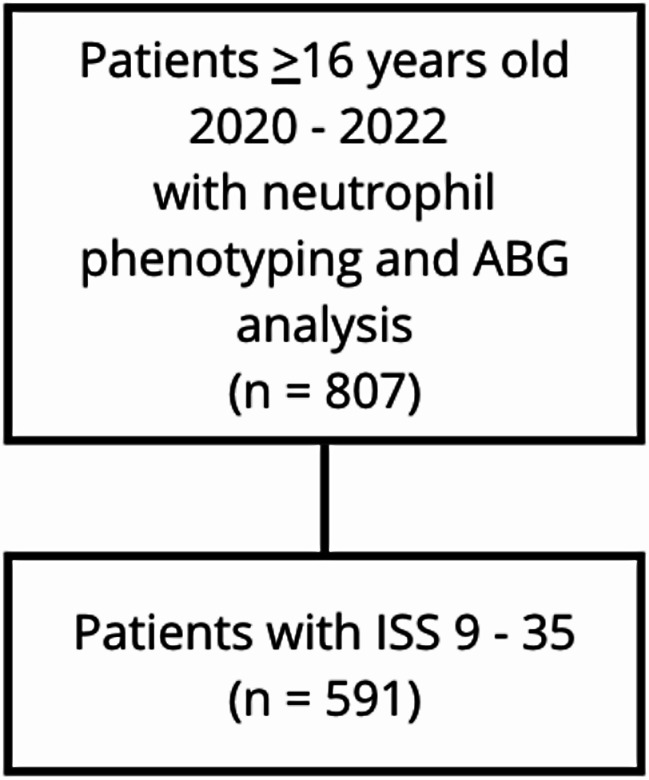
Flowchart of patients included in analyses. ABG Arterial Blood Gas

Between 2020 and 2022, a total of 807 patients *≥* 16 years old underwent neutrophil phenotyping and arterial blood gas analyses at ED presentation (Fig. 1).

### Baseline characteristics of all patients

The median age of the 807 patients who were included was 53 [IQR 32–70] years old, and 570 (70.6%) were male. The median ISS was 13 [IQR 9–21]. The most injured body region was the head in 298 (36.9%) patients. Hereafter, the most injured body region was the chest; 270 (33.5%, Table 1). The median, base excess was − 1.0 [IQR − 3.2–1.0] mmol/L and the median lactate level was 1.9 [IQR 1.4–2.8] mmol/L, the median number of banded neutrophils was 2.9 [IQR 1.0–11.1] x10^5^ cells/ml blood.

**Table 1:** Baseline characteristics

	*n*/median	(%)/IQR
*n*	807	
Age	53	[32 - 70]
Sex (M)	570	(70,6)
ISS	13	[9 - 21]
ISS *≥* 16	317	(39,3)
Hemodynamically unstable*	31	(3,8)
Glasgow coma scale *≤* 8	152	(18,8)
Injury per body region AIS *≥* 3		
Head	298	(36,9)
Face	14	(1,7)
Chest	270	(33,5)
Abdomen	64	(7,9)
Extremities	104	(12,9)
External	13	(1,6)
Number of banded neutrophils (×10^5^ cells/mL)	2.9	[1.0–11.1]
Base excess *≤* -4 (mmol/L)	163	(20,2)
Base excess (mmol/L)	-1.0	[-3.2 - 1.0]
Lactate *≥* 2.5 mmol/L	260	(32,2)
Lactate level (mmol/L)	1.9	[1.4 - 2.8]
Total length of stay (days)	5	[3–12]
ICU length of stay** (days)	3	[3–7]
Infectious complication	86	(10,7)
Mortality < 30 days	75	(9,3)

*Hemodynamic instability was defined as systolic blood pressure < 90 mmHg **Only patients admitted to the ICU

### Base excess levels, lactate levels and banded neutrophils in relation to ISS

**Fig. 2:**
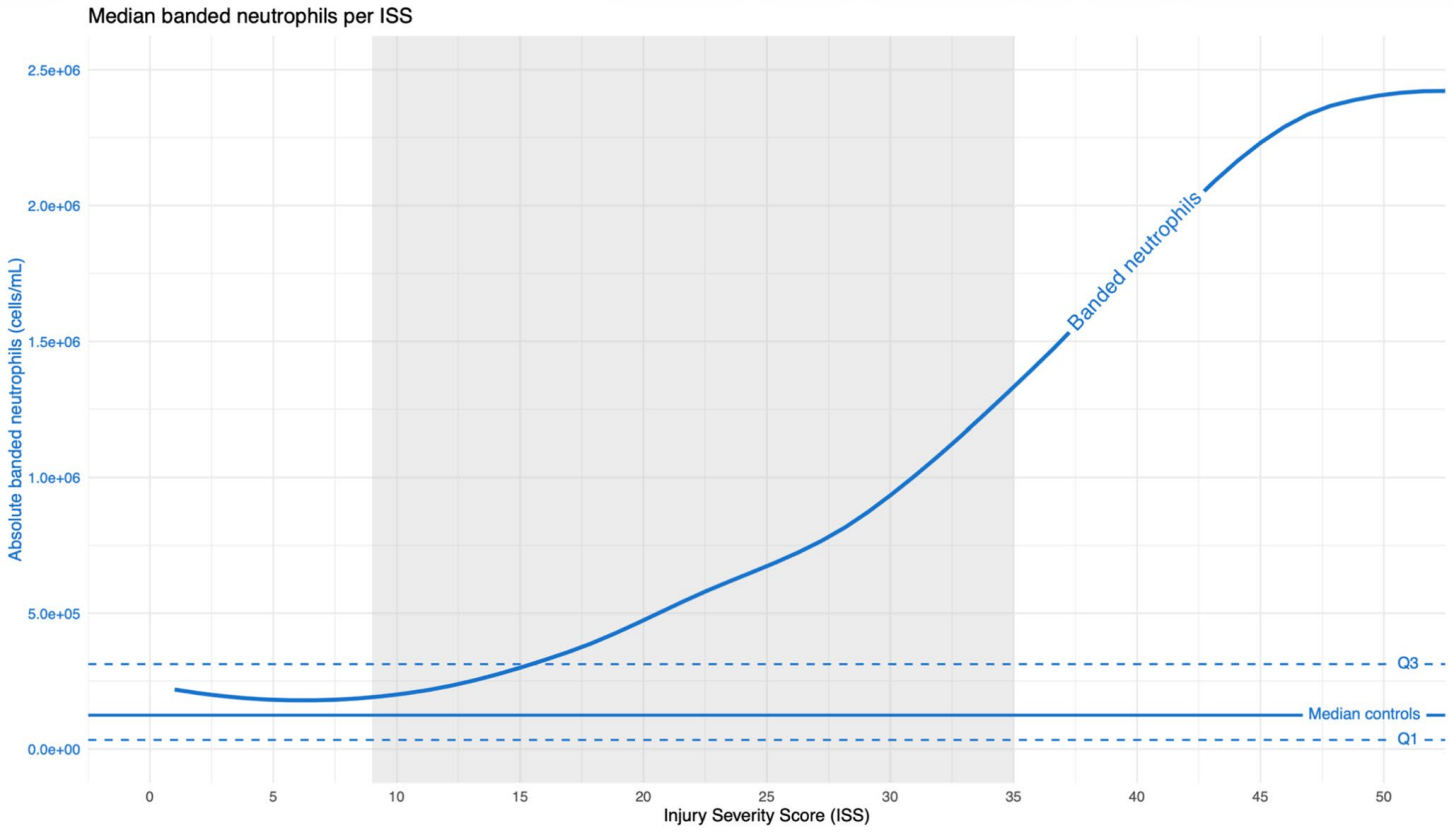
Median banded neutrophils per Injury Severity Score in relation to control values of patients with ISS *≤* 3

**Fig. 3:**
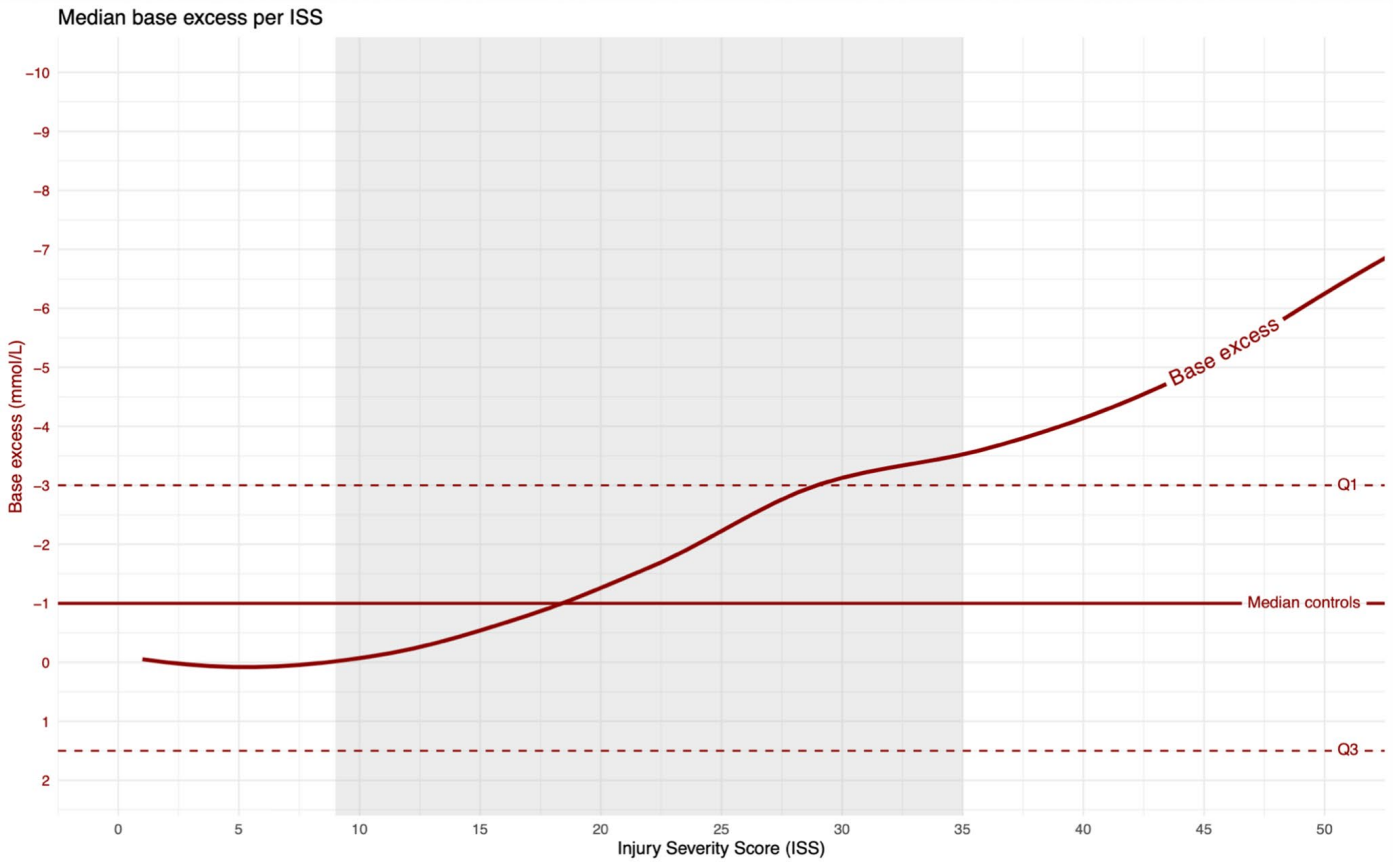
Median base excess per Injury Severity Score in relation to control values of patients with ISS *≤* 3

**Fig. 4:**
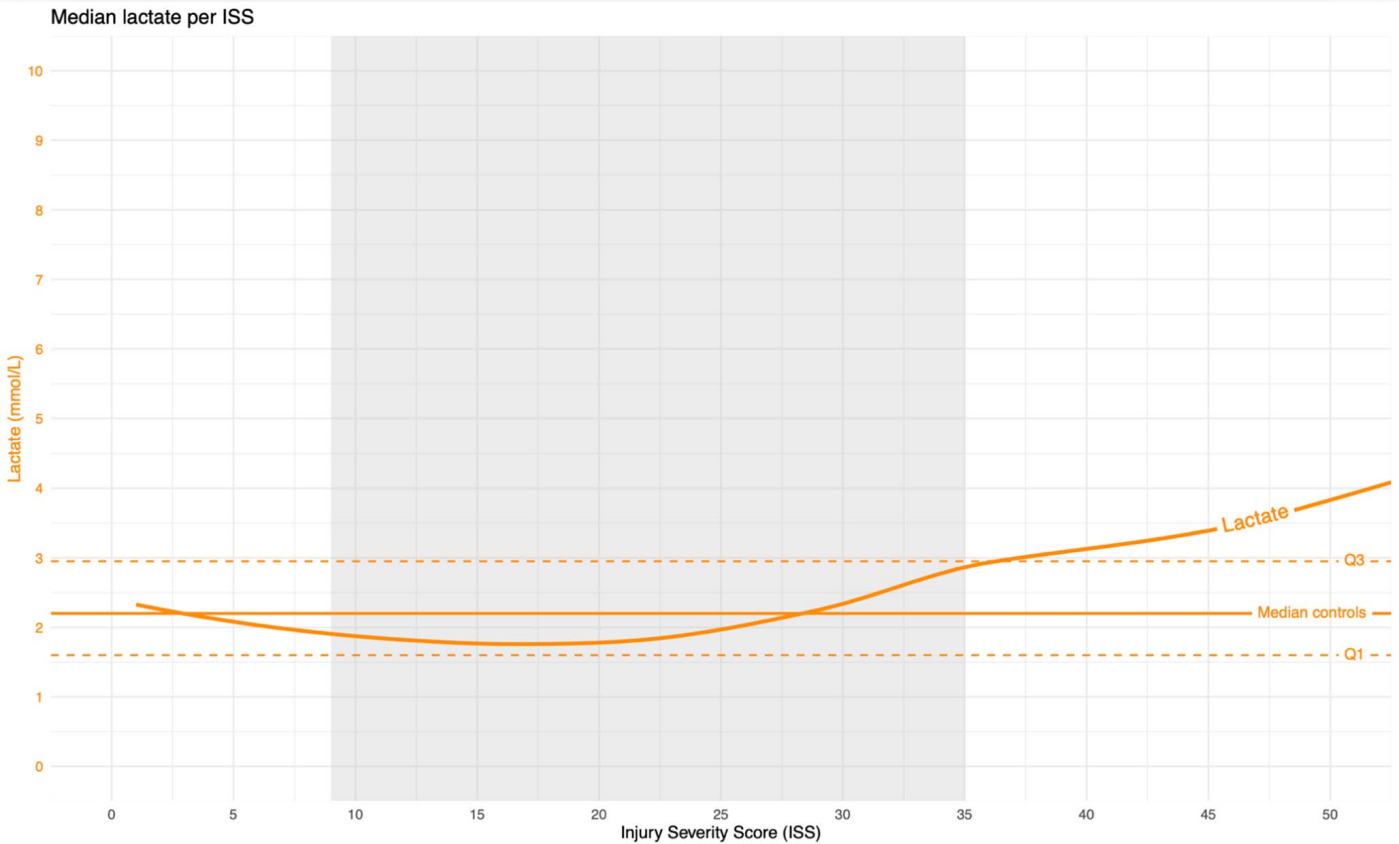
Median lactate levels per Injury Severity Score in relation to control values of patients with ISS *≤* 3

There were 591 patients with an ISS between 9 and 35. The relation of banded neutrophils, base excess and lactate to ISS were illustrated only in patients with an ISS < 50 to focus on midrange severely injured patients (Figs. 2, 3 and 4 and S1, S2, S3).

The median and IQR of control patients (ISS *≤* 3, without significant comorbidities, such as cardiac events, non-traumatic strokes, or epilepsy) were used as reference to determine the ISS at which parameter levels exceeded control values.

Banded neutrophils exceeded the upper interquartile range of controls at an average ISS of 17, whereas base excess and lactate levels both passed this threshold at an average ISS of 30 and 39, respectively. (Figures 2, 3 and 4; Table 2).

**Table 2:** Increase per parameter in trauma patients

	Fold change* between ISS 9–35	ISS level at which parameter passes Q3 controls
Banded neutrophils	6.7	17
Base excess	2.7	30
Lactate	1.5	39

Fold *increases in case of banded neutrophils and lactate, and *decrease in case of base excess between ISS 9 and ISS 35 were calculated by dividing the level at ISS 35 by the level at ISS 9. Ratios for base excess were calculated using a + 2 offset to account for values passing the zero-line

Spearman’s rank correlation analysis demonstrated significant associations between ISS and all investigated physiological parameters. Banded neutrophils showed a moderate positive correlation with ISS (ρ = 0.29, *p* < 0.001), and base excess demonstrated a moderate negative correlation (ρ = -0.26, *p* < 0.001). Lactate showed a weak but statistically significant positive correlation with ISS (ρ = 0.08, *p* = 0.02) (Table 3).

**Table 3:** Spearman’s rank correlation between parameters and Injury Severity Score

	Correlation coefficient (ρ)	*p*-value
Banded neutrophils	0.29	< 0.001
Base excess	-0.26	< 0.001
Lactate	0.08	0.02

Spearman’s correlation coefficients (ρ) and corresponding p-values showing the association between banded neutrophils, base excess, and lactate levels with the Injury Severity Score (ISS)

Finally, the total relative increase and decrease of each parameter was calculated in the patient group with an ISS of 9–35. Base excess showed a total 2.7 fold decrease between ISS 9–35, lactate showed a 1.5 fold increase, and banded neutrophils, a 6.7 fold increase (Table 2).

### Clinical outcomes

In multivariable regression analyses, higher numbers of banded neutrophil count were independently associated with hospital length of stay (β = 0.82, SE = 0.27, *p* = 0.003) (Table 4). No significant association was observed between numbers of banded neutrophils and ICU length of stay (β = 0.14, SE = 0.08, *p* = 0.10) (Table 4).

**Table 4:** Regression of banded neutrophils on clinical outcomes

	ß-coefficient/Odds Ratio	(SE) / [95% CI]	*p*-value
Hospital Length of stay	0.82	(0.27)	0.003
ICU length of stay	0.14	(0.08)	0.10
Infectious complication (yes/no)	1.22	[1.04–1.42]	0.01
Mortality (yes/no)	1.09	[0.90–1.32]	0.40

Logistic and linear regression on correlation between number of banden neutrophils and clinical outcomes. All analyses were corrected for age, gender and Injury Severity Score (ISS). Due to a strongly right-skewed distribution, absolute neutrophil counts were log-transformed before inclusion in regression models to improve model stability and interpretability

In logistic regression analysis, banded neutrophil count was significantly associated with the occurrence of infectious complications (OR = 1.22, 95% CI 1.04–1.42, *p* = 0.01) (Table 4). Banded neutrophils were not significantly associated with 30-day mortality (OR = 1.09, 95% CI 0.90–1.32, *p* = 0.40) (Table 4).

## Discussion

In this study, banded neutrophils rise above control values at lower injury severity scores compared to base excess and lactate (ISS 17 vs. 30 and 39, respectively). Furthermore, the increase in banded neutrophils for each increase in ISS point was greater than the fold change observed for base excess and lactate. This indicates a higher discriminative power for banded neutrophils in midrange trauma severity. As such, these findings indicate that neutrophil phenotyping may be of added value in clinical decision-making, especially in patients without clear physiological derangement observed in base excess and lactate levels, but with severe immunological distress.

### Relevance of the study for surgical decision-making

The decision between DCS and ETC is based on whether a patient is physiologically and immunologically too compromised to tolerate definite surgical intervention [3, 7, 24, 34]. In severely physiologically compromised patients, larger base excess and elevated lactate levels clearly favor DCS approaches [7]. In patients with very severe trauma (ISS > 35), it is often clear that a DCS approach is appropriately based on clinical findings, usually supported by physiological parameters such as declined base excess and elevated lactate levels reflecting the high injury burden. On the other hand, in patients with midrange severe trauma (ISS 9–35), base excess and lactate are less discriminative. Median values often remain within the normal range for an extended period (see Figs. 2, 3 and 4), making it difficult to assess the injury burden on these patients and thus hamper the judgement of interventional treatment strategy.

The present study proposes that in trauma patients with borderline base excess and lactate levels, neutrophil phenotyping may provide additional insight, especially on the immunological derangement. Inflammatory responses were already detectable at relatively low ISS levels and showed with a greater relative increase than the decrease in base excess or increase in lactate levels. It, thereby, provides an earlier indication of a compromised immune state in patients with still normal base excess or lactate levels. Therefore, the addition of neutrophil assessment supports a more informed surgical decision-making in these midrange severely injured patients.

### Factors that confound markers for injury

Several factors may influence the performance of biochemical markers. For example, elevated ethanol levels in the blood can affect both base excess and lactate values, causing artificially changed values due to altered metabolism and acid-base balance. This ethanol-induced shift can cause an overestimation of the base excess and lactate levels even in the absence of severe injury. This can lead to confounding data complicating correct interpretation [35].

In addition, some causes of traumatic injuries, such as falls resulting from acute coronary syndrome or epileptic seizures, may decrease base excess and elevate lactate levels. These changes are related to the underlying cause rather than the injuries itself [36–38].

How these above-mentioned factors affect neutrophil phenotypes must be investigated in future research. Given that the neutrophil compartment is primarily driven by DAMPs release following tissue damage, it is tempting to speculate that this process would not be influenced by factors such as ethanol [19].

On the other hand, neutrophils may be activated independently from injury by infection, allergy and/or autoimmunity and chronic inflammatory diseases such as diabetes mellitus and nonalcoholic fatty liver diseases [39–41]. Future research is needed to determine whether neutrophil status is an appropriate marker for injury severity in such populations.

### Association of banded neutrophils with clinical outcomes

Consistent with previous studies, an association was observed between the number of banded neutrophils and the occurrence of infectious complications [19]. The number of banded neutrophils measured immediately after traumatic injury reflects the magnitude of the post-traumatic neutrophil-driven inflammatory response [18, 19]. An excessive inflammatory response can disrupt immune balance, potentially resulting in trauma-induced immunosuppression [16–19]. This acquired immunodeficient state may increases susceptibility to infections and is therefore associated with a higher incidence of infectious complications.

### The effect of infectious/inflammatory complications on the diagnostic process in trauma patients

Incorporating immunological parameters such as neutrophil phenotype may be instrumental in further enhancing the quality of care for trauma patients. In recent years, significant advancements in trauma care have facilitated a shift towards more individualized, patient-tailored treatment strategies [42]. These improvements have contributed to a reduction and stabilization of mortality rates among severely injured trauma patients [42]. As mortality rates continue to decline, it becomes increasingly important to include complications alongside mortality as primary outcomes in trauma research. Complications contribute to higher morbidity, prolonged hospital stays, increased antibiotic use, and elevated healthcare costs [43]. Previous research has shown that an imbalanced post-traumatic inflammatory response may contribute to an increasing risk of infectious and inflammatory complications in trauma patients [19]. This post-traumatic inflammatory response is primarily driven by the release of DAMPs originating from tissue damage, releasing specific subsets of neutrophil phenotypes [19–21]. The neutrophil phenotyping in flow cytometry identifies a real-time immunological imbalance and has a predictive value of future risk of complications [18, 19].

### Improvements needed to achieve clinical applicability

Several conditions must be met before assessment of the inflammatory response can be integrated into clinical decision-making. These include rapid analysis, and safe reliable interpretation. The flow cytometry protocol used in this study requires only a single 4 mL blood sample and can be completed within thirty minutes without the need of professional personnel [44]. However, further research is necessary to develop automated quantitative metrics from flow cytometry results that can be objectively incorporated into the patient’s medical record.

### Limitations

One limitation of this study is its descriptive design, which inherently relies on the availability and accuracy of data in the patients’ medical records, for example in cases of incomplete documentation neutrophil phenotyping analyses. As patients were selected on availability of ABG analyses and neutrophil phenotyping data, this may have introduced bias. The available data on neutrophil phenotypes were collected prospectively.

## Conclusion

This study demonstrated a difference in dynamics for neutrophil phenotype, base excess and lactate levels after midrange severely injured trauma patients, indicating that neutrophil assessment might be of added value in surgical decision-making in these patients (ISS 9–35), where biochemical markers for physiological derangement are frequently only borderline elevated. Prospective studies investigating the integration of inflammatory markers into surgical decision-making are warranted.

## Supplementary Information

Below is the link to the electronic supplementary material.

Supplementary Material 1

## Data Availability

Restrictions apply to the availability of the data supporting the findings of this study, as they were used under license for the current study and are not publicly available. However, the data can be obtained from the authors upon reasonable request and with permission from University Medical Center Utrecht.

## Abbreviations

AIS: Abbreviated Injury Scale
DAMPs: Damage-Associated Molecular Patterns
DCS: Damage Control Surgery
DNTR: Dutch National Trauma Registry
ED: Emergency Department
ETC: Early Total Care
GCS: Glasgow Coma Scale
IQR: Interquartile ranges
ISS: Injury Severity Score
LOESS: Locally Estimated Scatterplot Smoothing
UPOD: Utrecht Patient-Oriented Database
