# Supplementary material for: Neutrophil phenotyping reflects injury severity when base excess and lactate remain normal
Source: Eur J Trauma Emerg Surg. 2026 Aug 12;52(1):242. doi: 10.1007/s00068-026-03285-3 (PMC13469382; doi:10.1007/s00068-026-03285-3)
Supplement: Supplementary file 1 [file 68_2026_3285_MOESM1_ESM.docx]

**Supplementary material**

**Figure S1** Median banded neutrophils per Injury Severity Score in relation to control values of patients with ISS ≤3, with datapoints

**Figure S2** Median base excess per Injury Severity Score in relation to control values of patients with ISS ≤3, with datapoints

**Figure S3** Median lactate levels per Injury Severity Score in relation to control values of patients with ISS ≤3, with datapoints
